# Book Reviews

**Published:** 1908-02

**Authors:** 


### BOOK REVIEWS.

---

PROGRESSIVE MEDICINE, VOL. IV, DECEMBER, 1907. A Quarterly Digest of Advances, Discoveries and Improvements in the Medical and Surgical Sciences. Edited by Hobart Amory Hare, M. D., Professor of Therapeutics and Materia Medica in the Jefferson Medical College, of Philadelphia. Octavo, 336 pages, with 30 engravings. Per annum in four cloth-bound volumes, $9.00; in paper binding, $6.00, carriage paid to any address. Lea Brothers & Co., Publishers, Philadelphia and New York.

Dr. Steele's monograph on Diseases of the Digestive Tract and Allied Organs brings the subject up to the present time, giving to the student, whether he be yet in his student years or aged and gray from life-long struggle with disease, a complete, clear epitome of all that has been added in recent times to our knowledge of normal and faulty digestion, and the means suggested for the alleviation of the latter condition.

Dr. John Rose Bradford's chapter on Diseases of the Kidneys comprises such subjects as Hydronephrosis, Renal Tumors in Children, Arteriosclerosis of the Renal Arteries, Renal Hematuria and Hydremia. Perhaps the subjects of most practical interest are Arteriosclerosis and Hypertension.

Dr. Joseph C. Bloodgood contributes the article on Surgery of the Extremities, fractures, dislocations, tumors, surgery of the joints, shock, anesthesia and infections. Under anesthesia are summarized the extremely valuable experiments of Crile in the Resuscitation of Animals apparently dead from Chloroform, Ether or Asphyxia, by artificial infusion with Adrenalin and Saline Solution, together with Crile's conclusions. Bier's induced hyperemia in chronic and acute infections is described and Scopolamine, Morphia Narcosis, Spinal Anesthesia and Local Anesthesia, with their comparative results, will be found in this chapter, which is profusely and excellently illustrated.

Dr. William T. Belfield contributes the article on Genito-Urinary Diseases, which will be found to contain a quantity of new material, as well as a careful study of the prostate gland and the comparative value of the different operations proposed for relief when diseased.

One turns eagerly into Dr. H. R. M. Landis' "Practical Therapeutic Referendum" to see what new thing may have developed in the neglected field of therapeutics—where, if ever, the knife of the surgeon is for a moment stayed and the physician breathes freely. Here there will be no disappointment, for Dr. Landis has the gift of choosing the good and refusing the worthless, a gift rare in these days of ephemeral remedies which bloom at the dawn and lie withered at sunset.

We have not yet thrown all drugs away, there are still left some to serve us when it is too late to advocate better hygiene, wiser diet and less alcohol, and when even psycho-therapeutics and vibratory machines fail us, we can find in just such pages as these of Dr. Landis', new remedies and new uses for old ones—Adrenalin for local use in Neuralgia, Apomorphine as a hypnotic and sedative and as a hopeful resource in Dipsomania, and Sodium Bi-Carbonate used in a novel way for gastric pain.

SUGGESTIVE THERAPEUTICS, APPLIED HYPNOTISM, PSYCHIC SCIENCE. By Henry S. Munro, M. D., Americus, Ga. Illustrated. C. V. Mosby, St. Louis, Mo.

The above work is a detailed explanation of how to apply suggestion efficaciously, with or without hypnotism. Dr. Munro gives his personal experience and clinical observations as proof of the value of suggestive therapeutics in the general practice of medicine. We all have a vague idea of the power of correctly applied suggestive measures, but few possess the practical essentials so necessary for their employment; much good may therefore be derived from a careful study of this book, which is written in a clear interesting style, and is easily assimiliable, as it has been prepared for the general practitioner, rather than for the specialists in this branch of science. We commend very highly this work and suggest that our readers procure a copy and give it a careful reading.

---

URIC ACID AND ITS CONGENERS: With special reference to its physical and chemical properties; its metabolism and accumulation in the organism, together with the disease processes arising therefrom and their etiological therapy. By George Abner Gilbert, M. D., Danbury Medical Printing Co., Danbury, Conn.

The fundamental principles underlying the accumulation of uric acid in the system is taken up in this book and carefully presented from its many view points. The table of contents shows the following headings to the chapters, which shows the scope of the work: It's place in Nature, historical review, physical characteristics, chemical properties, physiological processes, purin metabolism, evolutionary development, accumulation in the organism, disease processes, uricacidemic manifestations, rheumatic and gouty manifestations, sequelae and complications, urinary analysis, uric acid tests, Bright's disease, infants and children, women, ear, eye, nose and throat, traumatism, some usually unrecognized conditions, dental conditions, Haig's work, the food question, diebites, etiological therapy, current therapeutic methods effect of remedies in common use, concerning gout, therapy, the alkaline eliminants, Lithium and "final word."

